# First Report of the Italian Registry on Immune-Mediated Congenital Heart Block (Lu.Ne Registry)

**DOI:** 10.3389/fcvm.2019.00011

**Published:** 2019-02-28

**Authors:** Micaela Fredi, Laura Andreoli, Beatrice Bacco, Tiziana Bertero, Alessandra Bortoluzzi, Silvia Breda, Veronica Cappa, Fulvia Ceccarelli, Rolando Cimaz, Salvatore De Vita, Emma Di Poi, Elena Elefante, Franco Franceschini, Maria Gerosa, Marcello Govoni, Ariela Hoxha, Andrea Lojacono, Luca Marozio, Alessandro Mathieu, Pier Luigi Meroni, Antonina Minniti, Marta Mosca, Marina Muscarà, Melissa Padovan, Matteo Piga, Roberta Priori, Véronique Ramoni, Amelia Ruffatti, Chiara Tani, Marta Tonello, Laura Trespidi, Sonia Zatti, Stefano Calza, Angela Tincani, Antonio Brucato

**Affiliations:** ^1^Rheumatology and Clinical Immunology Unit, Department of Clinical and Experimental Science, ASST Spedali Civili, University of Brescia, Brescia, Italy; ^2^S.S.d.D.U. Immunologia, Allergologia, A.O. Ordine Maurziano di Torino, Torino, Italy; ^3^UO e Sezione di Reumatologia, Dipartimento di Scienze Mediche, Universita‘ degli Studi di Ferrara, Cona, Italy; ^4^Struttura Complessa Medicina Interna, ASST Papa Giovanni XXIII, Bergamo, Italy; ^5^Unit of Biostatistics, Biomathematics, and Bioinformatics, Department of Molecular and Translational Medicine, University of Brescia, Brescia, Italy; ^6^UO Complessa Reumatologia, Policlinico Umberto I- University La Sapienza, Rome, Italy; ^7^Anna Meyer Children's Hospital, University of Firenze, Firenze, Italy; ^8^Clinica di Reumatologia, Azienda Sanitaria Universitaria Integrata di Udine, Udine, Italy; ^9^UO Reumatologia, Dipartimento di Medicina Clinica e Sperimentale, Universita‘ di Pisa, Pisa, Italy; ^10^Istituto Ortopedico Gaetano Pini, University of Milan, Milan, Italy; ^11^Unità di Reumatologia, Dipartimento di Medicina, Università di Padova, Padova, Italy; ^12^Department of Obstetrics and Gynecology, ASST Spedali Civili and University, Brescia, Italy; ^13^Ginecologia e Ostetricia 1, Dipartimento di Scienze Chirurgiche, Università di Torino, Turin, Italy; ^14^Cattedra e Struttura Complessa di Reumatologia, Universita‘ degli Studi e AOU di Cagliari, Cagliari, Italy; ^15^Immunorheumatology Research Laboratory, Istituto Auxologico Italiano, Milan, Italy; ^16^UO Reumatologia, ASST Ospedale Niguarda, Milan, Italy; ^17^Rheumatology, IRCCS Policlinico San Matteo Foundation, University of Pavia, Padova, Italy; ^18^Dipartimento per la Salute della Donna, Bambino e Neonato, Fondazione Ospedale Maggiore, Milan, Italy; ^19^Dipartimento di Scienze Biomediche e Cliniche “Sacco”, Università degli Studi di Milano, Milan, Italy

**Keywords:** pregnancy, congenital heart block, neonatal lupus, outcome, risk factors, therapy

## Abstract

**Objective:** Neonatal Lupus (NL) is a rare syndrome caused by placental transfer of maternal anti-SSA/Ro and anti-La/SSB autoantibodies to the fetus. The rarity of this condition requires the establishment of multidisciplinary registries in order to improve our knowledge.

**Method:** Inclusion criteria in this retrospective study were the maternal confirmed positivity for anti-SSA/Ro and/or anti-SSB/La antibodies, and the presence of II or III degree congenital heart block (CHB) *in utero* or neonatal period (up to 27 days after birth).

**Result:** Eighty-nine cases of CHB were observed in 85 women with 88 pregnancies that occurred between 1969 and 2017. CHB was mostly detected *in utero* (84 cases, 94.2%), while five cases were observed in the neonatal period. A permanent pacemaker was implanted in 51 of 73 children born alive (69.8), whereas global mortality rate was 25.8% (23 cases): 16 *in utero*, five perinatal, and two during childhood. By univariate analysis, factors associated with fetal death were pleural effusion (*p* = 0.005, OR > 100; CI 95% 2.88->100 and hydrops (*p* = 0.003, *OR* = 14.09; CI 95% 2.01–122). Fluorinated steroids (FS) were administered in 71.4% pregnancies, and its use was not associated with better survival. Some centers treated all cases with fluorinated steroids and some centers did not treat any case. CHB was initially incomplete in 24 fetuses, and of them five cases of II degree block reverted to a lower degree block after treatments. Recurrence rate in subsequent pregnancies was 17.6% (3 out of 17). A prophylactic treatment was introduced in 10 of these 16 subsequent (58.8%) pregnancies, mostly with FS or high dose intravenous immunoglobulins.

**Conclusion:** This is the first report from the Italian Registry of neonatal lupus/CHB. The live birth rate was nearly 80%, with nearly two thirds of the children requiring the implantation of a pacemaker. The management of fetuses diagnosed with CHB was heterogeneous across Italian Centers. The registry at present is mainly rheumatological, but involvement of pediatric cardiologists and gynecologists is planned.

## Introduction

Neonatal lupus (NL) is a rare disorder mainly caused by the transplacental passage of maternal autoantibodies anti-SSA/Ro and/or anti-SSB/La (1, 2), usually during the second trimester of gestation (3, 4); these antibodies can reach the fetal heart, inducing inflammation (macrophage infiltration and giant cell formation), calcification, and fibrosis, which lead to aberrant signal conduction at the atrio-ventricular node. The most common manifestations are cutaneous or cardiac, while liver damage or cytopenia are less frequent. NL can occur in the offspring of mothers with a diagnosis of connective tissue disease (CTD), mostly Sjögren Syndrome (SS), or Systemic Lupus Erythematosus (SLE), but most cases are reported in asymptomatic women.

Cardiac involvement is usually irreversible and represents the most feared manifestation. It is characterized by advanced congenital heart block (CHB) (II or III degree) in an otherwise structurally normal heart.

Anti-SSA/Ro autoantibodies are found in ~85–90% of mothers of children with CHB (1), and prospective studies of pregnancies in anti-SSA/Ro positive patients estimated the risk of CHB to be 1–2% (5, 6). Recurrence rate in subsequent pregnancies is about 12–19% (1, 7, 8).

Several groups addressed the morbidity and mortality associated with CHB in different countries (9–13). Mortality ranges from 16 to 29%, whereas the rates of children receiving pacemaker vary from 50 to 79%, frequently within the first year of life. Studies are heterogeneous, also including cases not associated with maternal antibodies (9–14) (Table 1). The Italian Registry of Neonatal Lupus (Lu.Ne registry) was created to collect data also in Italy, supported by a grant from the Italian Society of Rheumatology. The aim was to determine the mortality and morbidity associated with CHB in an Italian cohort enrolling women with a confirmed positivity for anti-SSA/Ro and/or anti-SSB/La antibodies.

**Table 1:** Outcome of infants with CHB in the present study and in five large international series of cases (9–13).

	**Lopes et al. (9)**	**Eliasson et al. (10)**	**Izmirly et al. (11)**	**Levesque et al. (12)**	**Van der Berg et al. (13)**	**Present study**
N. of fetuses	57 with normal cardiac anatomy	175	325	202 *in utero* +12 in the neonatal period	56	84 *in utero* +5 in the neonatal period
Total mortality	13 (23%)	27 (15%)	57 (17.5%)	49 (23%)	9 (16%)	23 (25.8%)
Mortality *in utero*	6 (10%)	16 (9%)	18 (6%)	27 (13%)	8 (14.2%) (five additional cases of termination of pregnancies for various reasons	16 (17.9%)
Perinatal mortality	7 (14%)	10 (6.2%)	39 (12.7%) considered as 1 year after birth	8 (4%)	1 (2.3)	5 (5.6%)
PM cumulative prevalence	29 (56.7%)	102 (64%)	70% (the cumulative probability)	148 (79%)	30 (70%)	51 (69.8%)
Late onset cardiomiopathy	3 (5.6%)	8 (5.8%)	Four cases of heart transplantation	35 (18%)	6 (14%)	2 (2.2%)
Treated with FS	6 (10%)	67 (38%)	152 (47.8%)	79 (39%)	14 (27%)	60 (71.4%%)
Effects of FS	None	None on mortality; possibly reversal on II CHB	Possibly reversal on II CHB	None	None	None on mortality; possibly reversal on II CHB
Reversal of II degree CHB after FS	None	In 3/7 fetuses treated vs. 0/8 untreated	In 4/13 fetuses treated vs. 1/8 untreated	In 1/13 treated vs. 1/11 untreated	In 2/14 treated vs. 1/42	Five cases, all treated; see footnote^*^
Variables associated with death	Atrial rate < 120 bpm, ventricular rate < 55 bpm, hydrops,	Detection < 20 gw, ventricular rate < 50 bpm, hydrops, impaired left ventricular function	Earlier gestational age, lower ventricular rate, hydrops, EFE	Hydrops, prematurity (< 37 weeks gestation)	Not analyzed	Hydrops, pleural effusion
Survival rate at 10 years for a child born alive	NA	NA	86%	88%	NA	90%
Maternal anti-SSA/Ro antibodies	72%	80% of 162 pregnancies with documented antibody status	100%	99.5 %	89%	100%

* *1 case regressed from II degree to variable CHB (alternating between I and II degree), 2 from II to I degree and 2 regression from II degree to no CHB. Three out of the five fetuses were treated with a combined protocol composed by fluorinated steroids plus plasmapheresis plus IVIg, one received dexametasone plus plasmapheresis and one only dexametasone. NA, not available; CHB, congenital heart block; EFE, endocardial fybroelastosis; DCM, dilated cardiomyopathy; FS, fluorinated steroids; bpm, beats per minute*.

## Patients and Methods

### Study Cohort

The Lu.Ne registry was created in 2016, partially funded by the Italian Society of Rheumatology, after approval of the Institutional Review Board of the Coordinating Center in Brescia. Inclusion criteria were the maternal confirmed positivity for anti-SSA/Ro and/or anti-SSB/La antibodies and the presence of II or III degree CHB *in utero* or within the neonatal period (0–27 days after birth) (15) documented by electrocardiography and/or fetal echocardiography. For this study cases enrolled in the registry up to May 2018 were included. Medical records of pregnant women attending 11 Italian referral centers (mainly Rheumatology or Internal Medicine Departments, whose ethical committees approved the study) from 1969 to 2017 were retrospectively evaluated. In cases of variability of CHB grade, the most severe degree of CHB ever reached was considered for statistical analysis. This study was performed according to the principles of the Declaration of Helsinki with written informed consent from all subjects and was approved by the Ethic Committee of the Coordinating Center (approval number 2,417) and the participating centers.

### Data Collection and Definitions

Data were collected through an online electronic data sheet prepared in a Research Electronic Data Capture (REDCap) platform. Data obtained from medical files included: maternal age at birth, ethnicity, obstetrical history, the presence of a systemic connective tissue disease (CTD), an organ autoimmune disease or other known obstetrical risk factors.

The following data were collected about the fetus/child: the time of occurrence of CHB, the lowest prenatal ventricular and atrial heart rate, the presence of endocardial fibroelastosis (EFE), pericardial effusion, hydrops, dilated cardiomyopathy (DCM), valvulopathy or other anomalies (including ventricular and atrial-septal defects, intraauricular communication), treatment for CHB (dose and duration), maternal, and fetal outcomes. For children, we collected information on pacemaker implantation (PM), postnatal DCM, death, and other complications.

Fetal complications were defined according to common definitions (10–13). Atrioventricular block (AVB)-II° was defined as the intermittent mechanical dissociation of atrial and ventricular activation diagnosed by M-mode echocardiography and AVB-III° as the complete mechanical dissociation of atrial and ventricular activation diagnosed by M-mode (10, 13). AVB-I° was assessed only in the recent years, using pulsed Doppler echocardiography in the left ventricular outflow tract to record simultaneously mitral valve inflow and aortic outflow (mitral-aorta), from which the time delay from atrial systole to ventricular systole could be inferred. AVB-I° was diagnosed when this fetal mechanical Doppler PR interval was found to be >150 ms (16).

DCM was defined as increased size of the left ventricle or multiple chambers in the absence of chamber wall hypertrophy with associated decreased contractility on echocardiogram (11, 12); endocardial fibroelastosis as the presence of abnormal areas of echogenicity on the endocardial surface of the cardiac chambers and/or valve leaflets on echocardiogram or endocardial fibrosis on biopsy or autopsy. Hydrops fetalis was defined as an abnormal accumulation of fluid in at least two fetal compartments (11, 12).

In each center, autoantibodies tests were performed in a referral laboratory certified for diagnosis.

### Statistical Evaluation

Categorical variables were reported as proportion and/or percentage, while continuous variables as mean (±SD) values. Fisher's exact test or Chi-square test for categorical variables and Student's *t*-test or Wilcoxon-Mann-Whitney test for continuous variables were applied as appropriate. Multivariate analysis was not performed due to limited number of cases collected. *P* < 0.05 were considered significant and Odds Ratio (OR) with 95% Confidence Interval (95% CI) was indicated.

## Results

### Patients

By May 2018, the registry included 89 cases of CHB from 85 patients who had 88 pregnancies. The 85 women were Caucasian (*n* = 79, 92.9%), African (*n* = 3, 3.5%), Asian (*n* = 2, 2.3%), and Afro-Caribbean (*n* = 1, 1.2%) (Table 2). An organ-specific autoimmune disease was diagnosed in 12 women: autoimmune thyroiditis (*n* = 8, 9.4%), celiac disease (*n* = 3, 3.5%), multiple sclerosis (*n* = 1, 1.2%).

**Table 2:** Demographic information.

**Maternal demography**	***N* = 85 (%)**
**ETHNICITY**	
Caucasian	79 (92.9)
African	3 (3.5)
Asian	2 (2.3)
Afro-Caribbean	1(1.2)
**MATERNAL DIAGNOSIS AT CHB DETECTION**	
Undifferentiated Connective Tissue Disease	24 (28.2)
Sjögren's Syndrome	18 (21.2)
Systemic Lupus Erythematosus	4 (4.7)
Carriers of anti-SSA/Ro	24 (28.2)
Carriers of anti-SSA/Ro + anti-SSB/La	15 (17.6)
**ASSOCIATED ORGAN-SPECIFIC AUTOIMMUNE DISEASE**	
Autoimmune thyroiditis	8 (9.4)
Celiac disease	3 (3.5)
Multiple sclerosis	1(1.2)
None/Unknown	73 (85.6)
**AUTOANTIBODIES PROFILE**	
Anti-SSA/Ro	85 (100)
Anti-SSB/La	50 (58.8)

*CHB, congenital heart block*.

Sixty patients reported previous pregnancies, without previous documented cases of CHB, except for one case of cutaneous NL. When their first child with CHB was diagnosed, 46 mothers (54.1%) fulfilled the classification criteria for CTDs: undifferentiated connective-tissue disease (UCTD) (*n* = 24, 28.2%), SS (*n* = 18, 21.2%), SLE (*n* = 4, 4.7%), whereas the others were considered as anti-SSA/Ro carriers. Few cases of acquired cardiovascular risk factors were collected: two patients were smokers, one suffered from hypertension and obesity, and one had diabetes mellitus.

Four cases of multiple pregnancies were collected: three were spontaneous dichorionic biamniotic twins, with one affected, and one unaffected fetus for each pair. The other multiple pregnancy was a triplet gestation after *in vitro* fertilization: two out the three fetuses were affected by CHB (one III and one II degree) and one unaffected. The triplet pregnancy has already been described (17). Including the triplet pregnancy, three gestations that occurred after assisted reproductive technology procedures were collected.

All mothers were anti-SSA/Ro positive by inclusion criteria, and SSB/La antibodies were present in 58.8%. AntiRo52 status was available in 58.8% of the mothers, and all were positive.

The mean age at conception was 31.5 years (SD 5.3, range 22–42), 84 cases (94.4%) were diagnosed *in utero* at a median term of 21 gestational weeks (gw) (SD 4, range 17–38) and five (5.6%) were diagnosed in the neonatal period (15). CHB was initially incomplete in 24 fetuses (five with alternating II-III degree, two with alternating I-II degree, and 17 II degree). Considering the highest degree of CHB shown by the fetus/child, 71 (66 *in utero* and 5 neonatal) (79.8%) third-degree (complete) CHB, 18 (20.2%) second-degree CHB were included (Table 2).

### Fetal/Neonatal Outcomes

Among the 89 cases, 73 (82%) children were born alive at a mean gestational week (gw) of 35.3 (SD 3.0, range 28–41), 7 elective terminations of pregnancy (TOP) were performed at a mean term of 22 gw, and nine intra-uterine fetal deaths occurred at a mean term of 26 gw (Table 3). All the cases of TOP were CHB grade III. Table 4 reports the univariate statistical comparison of clinical and demographic features among survivors at birth and the deceased. By univariate analysis, factors associated with fetal death were pleural effusion (*p* = 0.005, *OR* > 100; CI 95% 2.88->100) and hydrops (*p* = 0.003, *OR* = 14.09; CI 95% 2.01–122).

**Table 3:** Outcomes of 89 cases of CHB.

**Pregnancy outcome**	***N* = 89 (%)**
Live birth	73 (82)
Intrauterine fetal death	9 (10.1)
Termination of pregnancy	7 (7.8)
**CHB DETECTION**	
*In utero*	84 (94.2)
**CHB GRADE**	
II degree	18 (20.2)
III degree	71 (79.8)
**OVERALL MORTALITY**	**23 (25.8)**
*In utero*	16 (18)
Neonatal	5 (5.6)
Childhood	2 (2.2)

**Table 4:** Comparison of clinical and demographic features among children born alive and fetuses died *in utero*.

	**Live birth *n* = 68 (%)**	**Deceased *n* = 16 (%)**	***p*-value**
***IN UTERO*** **DETECTED PATIENTS (84 CASES)**			
Maternal diagnosis of CTD	37 (54.4)	7 (43.7)	0.44
Non-Caucasian ethnicity	4 (5.4)	2 (12.5)	0.31
Maternal age at conception (SD)	31 (6.03)	32 (4.16)	0.76
Type of conception			
Spontaneous	64 (94.1)	16 (100.0)	0.73
Assisted reproduction techniques	4 (5.9)	0	
Timing of pregnancy			
Planned	21 (30.9)	6 (37.5)	0.767
Unplanned/unknown	47 (69.1)	10 (62.5)	
Gestational age at detection (gw) (SD)	22.8 (4.7)	20.7 (1.0)	0.27
Ventricular rate at nadir ≤ 50 bpm (*n* = 73)	21 (36.2)	6 (40)	0.78
Mean ventricular rate at nadir bpm (SD) (*n* = 73),	44.7 (27.9)	43.5 (30.8)	0.41
CHB grade (*n* = 84)			
II degree	16 (23.5)	2 (12.5)	0.3
III degree	52 (76.5)	14 (87.5)	
Impaired left ventricular function (*n* = 71)	5 (8.9)	3 (18.7)	0.35
Dilated cardiomyopathy (*n* = 74)	10 (12.6)	3 (27.3)	0.39
Hydrops (*n* = 82)	2 (3.0)	5 (31.2)	0.003^*^
Pleural effusion (*n* = 81)	0	3 (18.7)	0.005^**^
Pericardial effusion (*n* = 81)	8 (12.3)	5 (31.2)	0.12
Endocardial fibroelastosis (*n* = 81)	1 (1.5)	2 (13.3)	0.09
Intrauterine growth restriction (*n* = 75)	12 (19.3)	3 (23.1)	0.71
Oligohydramnios (*n* = 84)	5 (7.8%)	0	0.58

*CTD, connective tissue disease; gw, gestational week; bpm, beats per minute*;

* *OR, 14.09; CI 95% 2.01–122*,

** *OR > 100; CI 95% 2.88->100*.

The five cases diagnosed in the perinatal period or within the neonatal period (0–27 days after birth) occurred in the 1970–1980s: all these five newborns had III degree CHB; four of them received a pacemaker at a mean age of 7.2 years (range 2–18).

### Treatment

Prior to CHB identification, only a limited number of patients were receiving treatments (Table 5), in all cases for maternal disease: nine were treated with low dose aspirin (LDA), eight with not-fluorinated steroids, seven with hydroxychloroquine (HCQ), and one with immunosuppressive therapy (Table 5).

**Table 5:** Therapy before and after CHB detection.

	**Live birth *n* = 73 (%)**	**Deceased *n* = 16 (%)**	***p*-value**
**THERAPY BEFORE CHB DETECTION**			
LDA	6 (8.2)	3 (16.6)	0.36
Non-fluorinated steroids	6 (8.2)	2 (12.5)	0.67
Hydroxychloroquine	7 (9.6)	0	0.33
DMARDs	0 (0)	1 (6.2)	0.19
**MATERNAL THERAPY AFTER FETAL CHB DETECTION (*****n*** **=** **84)**			
	**Live birth** ***n*** **=** **68 (%)**	**Deceased** ***n*** **=** **16 (%)**	***p*****-value**
Any treatment	50 (73.5)	10 (62.5)	0.46
Fluorinated steroids	50 (73.5)	10 (62.5)	0.46
Intravenous Immunoglobulin	18 (26.4)	2 (12.5)	0.41
Plasma exchange	16 (23.5)	1 (6.2)	0.28
Other (beta-mimetics)	6 (8.9)	1 (6.2)	0.81
**CHB VARIATION DURING/AFTER THERAPY (*****n*** **=** **60)**			
	**Live birth treated** ***n*** **=** **50 (%)**	**Deceased treated** ***n*** **=** **10 (%)**	***p*****-value**
Regression	5 (10)	0	0.74
Progression	3 (6)	1 (10)	
Unchanged	38 (76)	8 (80)	
Unknown	4 (8)	1 (10)	

*LDA, low dose aspirin; DMARDS, immunosuppressive therapy*.

After CHB detection, fluorinated steroids (FS) were administered in 60 (71.4%) pregnancies, with a mean total duration of treatment of 9.5 weeks (range 4–18 weeks). Twenty steroid-treated fetuses (33%) received intravenous immunoglobulin (IVIg) and 17 (28.3%) received cycles with plasma exchange as well. Sixteen newborns received IVIg at birth.

Effects of treatments in the 60 treated pregnancies were analyzed and in the majority of the cases no variation in the progression of CHB was observed (46 cases, 76.7%) (Table 5).

CHB was initially incomplete in 24 fetuses, all of them were treated at least with FS; five cases of regression from grade II CHB was observed. In detail: one change occurred from II degree to variable CHB (alternating between I and II degree), two from II to I degree and two regression from II degree to no CHB. Three out of the five fetuses were treated with a combined protocol composed by fluorinated steroids plus plasmapheresis plus IVIg, 1 received dexamethasone plus plasmapheresis, and one only dexamethasone.

Fourteen cases of newborns small for gestational age, five cases of intrauterine growth retardation, four cases of olygohydramnios, one case of maternal hypertension were recorded in the 60 mothers treated with FS; these complications may be related to the treatment with FS, particularly olygohydramnios and hypertension.

### Postnatal Outcomes

Among the 73 live births, five newborns died within 10 days after birth (Table 6). These five children were born prematurely and in four cases death occurred even if a pacemaker was placed at birth.

**Table 6:** Pregnancy outcome and postnatal follow-up in pregnancies ended with a live birth.

**Pregnancy outcome**	**Live birth *n* = 73 (%)**
Medium gestational week of delivery (SD) (*n* = 70)	35.3 (3.0)
Delivery (*n* = 73)	
Cesarean section	58 (82.3)
Vaginal	12 (17.1)
unknown	3 (6)
Preterm deliveries < 37 weeks	53 (72.6)
Preterm deliveries < 34 weeks	26 (35.6)
Sex (*n* = 71)	
Female	44 (62)
Male	27 (38)
Medium weight at birth (grams) (SD) (*n* = 69)	1776.5 (523.5)
Medium length (cm) (SD) (*n* = 40)	40.5 (5.8)
APGAR (1–10) (*n* = 55)	8.5 (1)
DCM at birth (*n* = 72)	5 (7.0)
**POSTNATAL OUTCOME**	
At birth/Neonatal PM implantation	29 (39.7)
Neonatal death	5 (6.8)
Infant/childhood PM implantation	22 (30.1)
Infant/childhood death	2 (3.1)
Overall PM pacing	51 (69.8)
Overall mortality	23 (25.8)

*DCM, dilated cardiomyopathy; PM, pacemaker*.

Out of the remaining 68 children, two died later, one due to late onset DCM at the age of 21 months after a PM placed at birth, and 1 at the age of 6 years for a sudden death, probably due to a thrombotic event, however autopsy was not performed. Another child underwent cardiac transplantation at the age of 17 months for late onset DCM in 2003, and at present he is doing well.

Overall DCM was recorded in six cases at birth, while two cases of late onset DCM were observed (2.2%) (see Table 1). All the children with DCM were permanently paced, and two of them died (25%).

Overall a PM was placed in 51 of the 73 children born alive (69.8%): 19 (37.2%) at birth, 10 (19.6%) within the first month of life, 11 (21.5%) within the first year of life, and 11 later (21.5%). Within the first year of life, more than 50% of the surviving children were paced (40 children, 54.8%).

### Recurrence

After the index pregnancy, 14 women had 17 subsequent pregnancies (reviewed in Table 7): three were complicated by a CHB therefore the recurrence rate in our cohort was 17.6%. Nine patients received treatments during 10 pregnancies (58.8%): hydroxychloroquine in 1, IVIg alone in 1, not-fluorinated steroids (for maternal indication) alone in 3, not-fluorinated steroids and IVIg in 3, IVIg and HCQ in 1, and IVIg with plasmapheresis and fluorinated steroids in 1. Non fluorinated steroids and HCQ were administered before pregnancy, fluorinated steroids were introduced at conception in two cases, and IVIG and plasmapheresis were started from week 12 (see Table 7 for details).

**Table 7:** Subsequent pregnancies after an index pregnancy complicated with CHB: treatment and pregnancy outcomes.

	**Index pregnancy outcome**	**Maternal diagnosis**	**Year of pregnancy**	**Fetal ECHO**	**Treatment**	**Pregnancy outcome**	**Pregnancy complications**
Pt 1	CHB III degree, born alive	SS	2005	yes	Prednisolone 28 mg/w IVIg 400mg/kg every 3 w between 12 and 24th gw	Born alive, without CHB	no
Pt 2	CHB III degree, neonatal detection, infant death	SS	1978	yes	no	Neonatal CHB III degree	no
Pt 3	CHB III degree, born alive	Carrier anti-SSA/Ro	2014	yes	no	Born alive, without CHB	no
Pt 4	CHB III degree, infant death	UCTD	2003	yes	no	Born alive, without CHB	no
			2006	yes	no	Born alive, without CHB	no
Pt 5	CHB II degree, born alive	SS	2007	yes	IVIg 400 mg/kg every 3 w between 12 and 24th gw HCQ 200 mg/daily	Born alive, without CHB	no
Pt 6	CHB III degree, TOP	UCTD	2006	yes	Prednisone 35 mg/w; IVIg 400 mg/kg every 3 w between 12 and 24th gw	Born alive, without CHB	Polyhydramnios
			2008	yes	Prednisone 25 mg/w; IVIg 400 mg/kg every 3 w between 12 amd 24h gw	Born alive, without CHB	Maternal hypertension
Pt 7	CHB III degree, TOP	SS	2002	yes	Prednisone 35 mg/w;	Born alive, without CHB	no
			2009	yes	no	Born alive, without CHB	no
Pt 8	CHB III degree, intra-uterine fetal death	Carrier SSA/Ro +SSB/La	2012	yes	Betametasone 28 mg/w, IVIg 1 g/kg every 2 w for 13w, Plasmapheresis for 14 w	CHB II degree	Olygo-anydramnios
Pt 9	CHB III degree, born alive	UCTD	1999	yes	Betametasone 10 mg/w	Born alive, without CHB	IUGR, maternal hypertension
Pt 10	CHB III degree, intra-uterine fetal death	Carrier SSA/Ro +SSB/La	2001	yes	Dexametasone 28 mg/w	CHB III degree	PM at birth
Pt 11	CHB III degree, TOP	UCTD	2007	yes	IVIg400 mg/kg every 3 w between 12 and 24th gw	Born alive, without CHB	
Pt 12	CHB III degree, born alive	Carrier SSA/Ro +SSB/La	2015	yes	no	Born alive, without CHB	
Pt 13	CHB III degree, intra-uterine fetal death	UCTD	2014	yes	HCQ	Born alive, without CHB	Oligohydramnios

*We did not include in the table one case that ended with an early termination of pregnancy required by parents at 11 gw. ECHO, echocardiography; HCQ, hydroxychloroquine; TOP, termination of pregnancy; PM, pacemaker; UCTD, undifferentiated connective tissue disease; SS, Sjögren Syndrome; IUGR, intra-uterine growth restriction; IVIg, intravenous immunoglobulin; HCQ, hydroxychloroquine*.

Adverse events possibly related with a prolonged use of steroids (maternal hypertension, intra-uterine growth restriction, oligohydramnios) occurred in three. The recurrence rate was not statistically different in mothers who received steroids compared to those who did not (28.6 vs. 11.1%, respectively, *p* = 0.55), but the numbers are low. All the three recurrences of CHB occurred after an index pregnancy complicated with fetal or neonatal death due to a complete CHB.

### Maternal Follow-Up

At the time of index pregnancy, 39 patients were considered as asymptomatic autoantibodies carriers. Two years after the latest pregnancy, 11 patients of them developed signs/symptoms that fulfilled the criteria for connective tissue disease: six cases of UCTD and five of SS. In six patients, a chronic treatment was required: oral steroids in four, HCQ in three, and methotrexate in one.

## Discussion

This paper describes the first data from the Italian Registry of neonatal cardiac lupus syndrome, including 89 retrospective cases of CHB associated with anti-SSA/Ro and/or anti-SSB/La antibodies. This registry was created in order to collect the cases diagnosed and treated in different Centers, some of them with a longstanding interest in this rare condition. Although some of the cases included in this registry have been already published (17–20), this remains the first effort to analyze all the data as a collaborative national study.

The results that were obtained are in many aspects in line with the published large retrospective studies (Table 1) (9–13). The number of cases of complete and incomplete CHB (79.8 vs. 20.2%) and the cumulative probability of pacemaker implantation, almost 70%, were very similar to already published data (1, 9–13) (see Table 1).

The risk of fetal mortality in the present cohort was 18% and the overall mortality was 25.8%, slightly higher in our cohort than reported in other publications (see Table 1). On statistical analysis, several risks factors that were associated or had a trend toward an increased risk for mortality were confirmed. The presence of hydrops and fetal serositis are well established risk factors for adverse outcome, confirmed in several previous papers (1, 11, 12). No other risk factors were identified in our cases, in particular fetal mortality was not associated with a maternal diagnosis of SLE or SS at the time of pregnancy or a specific ethnicity as previously reported (11).

Some confusion existed in the past on the definition of “congenital” heart block, with some cases detected after birth; for this reason a multidisciplinary group proposed to define congenital heart block as an atrioventricular block diagnosed *in utero*, at birth or within the neonatal period (15) and in the present report five cases were diagnosed after birth.

In our registry data on subsequent pregnancies after a case of CHB were also collected; recurrence rate of CHB was 17.6%, strikingly similar to what found (17.4%) (7) in the American Research Registry for Neonatal Lupus; in our registry all the three fetuses with recurrent CHB were born alive.

Till date, the management of CHB remains very controversial and there are no generalized recommendations on how to treat CHB or if a prophylactic treatment is required during pregnancy. Various treatment approaches have been reported, including steroids, plasmapheresis, IVIg, several immunosuppressive agents, and hydroxychloroquine (21). Fluorinated steroids (FS) could cross the placenta because they are only partially inactivated by 11ß-hydroxysteroid dehydrogenase complex expressed in syncytial trophoblast cells and have satisfactory bioavailability to the fetus (22), and are the drugs with the largest clinical experience. Side effects of high dose FS during pregnancy may be important: increased blood pressure, osteopenia, osteonecrosis, susceptibility to infections, gestational diabetes, premature rupture of the membranes and olygohydramnios. In the present study 60 women were treated with FS: olygohydramnios occurred in 6.6% of cases, intrauterine growth retardation in 8.3% and hypertension in 1.7%.

Retrospective data over a wide time span ranging from the 1970s through 2017 were collected in the present study, therefore the treatment strategies were very heterogeneous. Steroids resulted as the most used drugs, reaching the highest rate compared with other registries (see Table 1) and this result confirms that there is no consensus regarding treatment with steroids. Moreover, in many occasions, it depends on the historical approach followed in the single center (23): some centers treated no patients, irrespective of the fetal status, whereas in others hospitals FS were used almost in all cases. The most consistent data on the possible efficacy in CHB were published by Jaeggi et al. (24) in 2004. The authors reported a higher one-year survival rate and less complications or features associated with NL in 21 treated complete CHB compared with 11 patients who did not receive FS. This study, however, displays some limitations. Firstly, the authors compared fetuses from two different eras: the historical cohort from 1990 to 1996 did not receive steroids, whereas all fetuses between 1997 and 2003 were treated. A second important limitation was the higher rate of risk factors for a poor prognosis present in the untreated cohort. Subsequent works did not confirm these findings (10–12, 25). In fact, we also did not find any significant differences on fetal mortality between the groups treated and not with FS, which is consistent with the large international series.

In particular Izmirly et al. (25) compared 71 fetuses with isolated CHB who received FS within 1 week of detection with 85 who received no treatment and evaluated the development of EFE, dilated CMP, hydrops, mortality, and PM implantation. These authors observed that FS did not significantly prevent development of disease beyond the atrio-ventricular node [adjusted Hazard Risk (HR) = 0.90; *p* = 0.77], nor reduce mortality (*HR* = 1.63; *p* = 0.47), or forestall/prevent PM implantation (*HR* = 0.87; *p* = 0.53), so they concluded that no evidence supports fluorinated steroids to prevent disease progression or death in isolated CHB.

Another possible indication for the use of FS is for the prevention of the evolution from incomplete to complete CHB. Whereas, complete CHB is considered irreversible, regression from incomplete block after treatment has been described (10, 11, 26–28). In our cohort an improvement was observed in five cases, all treated with FS and three treated with a combination therapy recently published (29). In brief, in that paper (29) the authors wanted to summarize the possible effects of each single procedure: they demonstrated that plasmapheresis could remove anti/SSA-Ro autoantibodies (30), FS could reduce local inflammation and IVIg could limit the effects of autoantibodies. They used this approach in 12 patients with second or third degree CHB. No variation occurred in the six cases with complete CHB, whereas an improvement occurred in 50% of second degree CHB. The authors reported no side effects in the fetuses or in the mothers, proposing this combination therapy as a therapeutic option in second degree CHB. Unfortunately, since such improvement has been observed also in the absence of any treatment (12) or only with FS, it is not possible to draw any definite conclusion. The recent paper by Cuneo et al. (28) underlines as timing may very relevant for a possible therapeutical windows.

Several hypotheses have been proposed showing the potential usefulness of IVIG to prevent cardiac tissue damage: firstly increasing the elimination of maternal autoantibodies through IVIG saturation, secondly decreasing placental transport of autoantibodies through FcγRn leading to the modulation of inhibitory signaling on macrophages, with consequent reduction of the inflammatory response and fibrosis. This explain the patients treated during pregnancies and the 16 newborns treated immediately after birth (31–33).

There are no specific guidelines for the prevention of recurrence of CHB in subsequent pregnancies and this explains the extreme heterogeneity of treatment that was observed in this cohort, ranging from only clinical and echocardiography monitoring to combined therapies during pregnancy. Non-fluorinated steroids do not cross the placenta and would not be useful at all. Intravenous immunoglobulin has been proposed in the prevention of recurrence in small case series and in two prospective studies that were performed in Europe and in United States (34, 35) with a similar protocol (400 mg/kg every 3 weeks from 12 to 24 gw). Four of our cases were included in the European trial. Both the studies were terminated early because of an unchanged prevalence of recurrence and it was concluded that IVIg at the proposed dose was ineffective at reducing the recurrence rate of cardiac NL.

In the last years, the use of HCQ was shown to be a possible approach to the secondary prevention of the recurrence of CHB. Retrospective analysis from an international cohort (36) reported a higher recurrence rate in pregnancies not treated with HCQ compared with those treated with HCQ. In our study only a limited number of pregnancies were exposed to HCQ not allowing any possible further analysis. However, since the use of HCQ is compatible with pregnancy (37) and is generally a well-tolerated drug, it may be proposed in patients with known antibody positivity.

Our study has several limitations. Data were collected retrospectively and in some pregnancies not all of the data were available, which limits the power of our statistical analysis. It is well established that the distinction between II and III degree AV block *in utero* may be difficult, problematic and time consuming and, when revised centrally, some diagnoses of II degree might be reclassified as III degree and viceversa (13). For this paper it was not possible to reassess the diagnosis centrally therefore some complete CHB could be misdiagnoses as incomplete (13). CHB cases whose mothers were anti-SSA/Ro negative were not included (17). This first report of the registry is mainly driven by rheumatological centers, and some geographical Italian regions are not represented; only the centers whose ethical committees approved the study enrolled cases for this initial analysis. This peculiarity might also explain why in our registry the majority of the mothers already had a diagnosis of CTD at the time of the index pregnancy, an evidence that differs from other experiences. The syndrome of course requires a multidisciplinary approach, not only for the clinical management of each case but also for the systematic collection of the data and their analysis. Pediatric cardiologists and gynecologists play a fundamental role in the management of this condition, and it is planned to involve them in further collections and analyses of data.

In conclusion, this is the first preliminary report of the data from the Italian Registry of neonatal cardiac lupus syndrome, that was established in 2016. Italian centers showed an heterogenous pattern of management of CHB fetuses, with some centers treating all cases with FS and some centers treating no cases. The establishment of this registry might help to share the data, to make more homogenous the management of this rare condition and to stimulate further multidisciplinary studies.

## Author Contributions

AB, AT, MF, and LA: designed the study. MF, SC, VC, and LA: created the registry on RedCap platform. MF, TB, AB, SB, FC, RC, SD, ED, EE, FF, MGe, MGo, AH, AL, LM, AMi, PM, AM, MMo, MMu, MeP, MaP, RP, VR, AR, CT, MT, LT, and SZ: evaluated the patients. MF, TB, ABo, SB, FC, ED, EE, MGe, AH, AMi, MeP, MaP, VR, CT, BB, and MT: recruited the patients. MF, LA, AB, and AT: wrote the manuscript. All the co-authors reviewed the manuscript.

### Conflict of Interest Statement

The authors declare that the research was conducted in the absence of any commercial or financial relationships that could be construed as a potential conflict of interest.
